# Mesenchymal Stromal Cells at the Interface of Hemostasis and Immunothrombosis

**DOI:** 10.3390/biology15090728

**Published:** 2026-05-03

**Authors:** Luca Bonanni, Nicola Ferri, Paolo Simioni

**Affiliations:** 1 Department of Medicine, Ospedale dell’Angelo, 30174 Venice, Italy; 2 Department of Medicine, University of Padua, 35123 Padua, Italy; nicola.ferri@unipd.it (N.F.); paolo.simioni@unipd.it (P.S.)

**Keywords:** mesenchymal stromal cells, immunothrombosis, tissue factor, thrombin generation, extracellular vesicles, complement activation, platelet interaction, hemocompatibility, cell therapy, coagulation

## Abstract

Mesenchymal stromal cells are increasingly used for their immunomodulatory and regenerative properties, yet their interaction with the hemostatic system remains incompletely understood. This review examines the mechanisms through which these cells influence coagulation within the broader framework of immunothrombosis. Evidence from in vitro studies, animal models, and early clinical observations indicates that mesenchymal stromal cells can promote thrombin generation through tissue factor expression and phosphatidylserine exposure, while also engaging complement pathways, platelets, and innate immune responses. Counter-regulatory mechanisms, including adenosine-mediated platelet inhibition and immune reprogramming after cellular clearance, contribute to a context-dependent biological effect. Functional assays, rather than tissue factor expression alone, appear necessary to estimate the effective procoagulant potential of these products. Clinical data suggest that major thrombotic events remain uncommon, although subclinical activation of coagulation pathways may occur. The hemostatic impact of mesenchymal stromal cells depends on multiple variables, including cell source, dose, route of administration, and host inflammatory status. The available evidence supports a working model in which early coagulation and complement activation may be followed by immune modulation, supporting integrated strategies to optimise both safety and therapeutic efficacy. A central conclusion is that tissue factor, although mechanistically necessary for MSC-associated procoagulant activity, is not by itself an independent predictor of clinical thrombotic risk; the effective coagulation response also depends on phosphatidylserine exposure, membrane context, and host inflammatory conditions.

## Simple Summary

Mesenchymal stromal cells are being studied as treatments for many diseases because they reduce inflammation and support tissue repair. After intravenous administration, they come into immediate contact with blood and may activate linked coagulation and innate immune pathways. In this review, we describe how these cells interact with clotting factors, platelets, and immune cells. These interactions are neither uniformly harmful nor uniformly beneficial; they depend on cell type, dose, and patient condition. In some settings, processes that increase clotting risk may also contribute to control of inflammation and support of healing. Understanding this balance is important for making cell therapies safer and more effective. Future strategies should not aim to block these reactions completely, but to measure and control them in a targeted way. Functional hemocompatibility testing of each cell batch before infusion, used as a product-release criterion, could identify preparations with unacceptably high procoagulant activity and complement the phenotypic markers currently used.

## 1. Introduction

Mesenchymal stromal cells (MSCs) have been widely investigated for their regenerative and immunomodulatory properties. Clinical interest has focused on their capacity to modulate inflammatory responses and to support tissue repair in conditions such as systemic injury or immune dysfunction. Systemic administration introduces biologically active cells into the circulation, where they may interact with the immune system and with components of the hemostatic network.

Clinical applications of MSCs span a broad range of indications in which immunomodulation and tissue repair are the principal therapeutic targets. Controlled trials and compassionate-use programmes have explored MSC administration in steroid-refractory acute graft-versus-host disease, acute respiratory distress syndrome including COVID-19–associated lung injury, Crohn’s disease fistulae, spinal cord injury, osteoarthritis, and acute myocardial infarction, with additional programmes in acute kidney injury, liver failure, and diabetic nephropathy [1]. As clinical use has expanded, the hemocompatibility profile of intravenously administered MSCs has become an active area of debate. Concerns regarding tissue factor–driven procoagulant activity, instant blood-mediated inflammatory reaction, and thromboembolic complications in high-risk recipients coexist with large safety meta-analyses reporting low rates of major thrombotic events, producing a discrepancy between mechanistic preclinical signals and aggregate clinical outcomes that the present review examines.

In many clinical settings in which MSCs are proposed or administered, systemic inflammation and endothelial activation coexist with alterations of coagulation and platelet activation. Coagulation and innate immunity do not simply occur in parallel; they may reinforce each other through interconnected feedback pathways involving tissue factor (TF), thrombin generation, fibrin formation, myeloid cells, complement activation, and inflammatory mediators [2,3,4,5]. Localized stimuli, such as exposure to a procoagulant surface or a transient inflammatory signal, may expand within the microcirculation once vascular homeostasis has been primed by inflammation [3,4,5].

This interface between innate immunity and coagulation has been described as “immunothrombosis”, a process in which intravascular thrombus formation is largely driven by innate immune mechanisms. In physiological settings, the response may contribute to host defense by restricting pathogens within the microcirculation. Under pathological conditions, the same mechanisms may evolve toward a dysregulated response associated with diffuse microthrombosis, disseminated intravascular coagulation, and organ injury [3,4,5,6,7]. Attention therefore shifts from the concept of thrombotic risk alone toward a broader question: how a biological intervention interacts with a system in which inflammatory and hemostatic pathways are tightly interconnected [2,3,4,5]. This perspective may help explain the variability of hemostatic effects reported after MSC administration. It may also support the development of safety criteria that account for product characteristics and for the clinical context in which cells are administered.

The evidence discussed in this review derives from a combination of in vitro studies, animal models, and limited clinical observations, with variable consistency across experimental and clinical settings.

The review is framed as a narrative synthesis rather than a systematic appraisal. Literature was retrieved through PubMed, using combinations of the following terms: mesenchymal stromal cells, mesenchymal stem cells, tissue factor, CD142, procoagulant activity, thrombin generation, instant blood-mediated inflammatory reaction, complement, neutrophil extracellular traps, regulatory T cells, hemocompatibility, and thrombogenic risk. The retrieval window extended from 2005 to 2025, with no restriction on study design. Preclinical in vitro, ex vivo whole-blood, animal, and clinical studies were all eligible. Priority was given to mechanistic studies, systematic reviews and meta-analyses, and clinical trials with coagulation-related endpoints. Evidence quality was evaluated qualitatively rather than through a PRISMA-equivalent flow, consistent with the narrative format. Clinical trials without hemostatic or coagulation-related endpoints were included only when they provided safety signals relevant to immunothrombotic mechanisms.

Previous reviews have addressed TF-driven procoagulant activity, IBMIR, complement opsonization, hemocompatibility assays, and the dose and source dependency of MSC thrombogenicity [8,9,10,11]. The present work does not aim to duplicate that conceptual territory. Its added value lies in the articulation of a hypothetical “cell sacrifice” mechanism, in which complement activation and phagocytic clearance are reinterpreted not only as obstacles to therapeutic efficacy but also as possible functional components of the immunomodulatory programme. A second, more tentative angle concerns the possible participation of regulatory T cell biology in the resolution phase of MSC-driven immunothrombosis; this is developed explicitly as a speculative extension rather than as a demonstrated mechanism.

This perspective also provides the organising logic of the review. The early contact between MSCs and blood is first analysed through the mechanisms that may initiate or amplify intravascular activation, including tissue factor expression, phosphatidylserine-dependent coagulation complex assembly, platelet engagement, extracellular vesicle activity, complement activation, and NET-related responses. The review then moves from this early procoagulant and inflammatory interface to the subsequent fate of infused cells, focusing on complement deposition, cellular injury, and phagocytic clearance as possible links between blood-mediated activation and immune regulation. On this basis, the final part of the manuscript proposes a biphasic interpretative framework and defines a limited set of testable predictions.

## 2. MSCs as Dynamic Modulators of the Immunothrombotic Axis

MSCs are a heterogeneous population of multipotent adherent progenitor cells defined by a consensus phenotype (positivity for CD73, CD90, and CD105; absence of hematopoietic and endothelial markers), plastic adherence, and trilineage differentiation toward osteogenic, adipogenic, and chondrogenic lineages. They can be isolated from adult and perinatal tissues including bone marrow, adipose tissue, umbilical cord and Wharton’s jelly, placenta and decidua, dental pulp, and liver, each source carrying distinctive transcriptional profiles and functional properties [1,12]. The therapeutic rationale rests primarily on immunoregulatory activity rather than on engraftment. Through contact-dependent mechanisms and paracrine signaling, MSCs suppress T-cell proliferation, promote regulatory T-cell expansion, polarize macrophages toward an anti-inflammatory M2 phenotype, and release soluble mediators such as indoleamine 2,3-dioxygenase, prostaglandin E2, TGF-β, and extracellular vesicles that modulate innate and adaptive immune responses [12]. This immunoregulatory repertoire places MSCs at the interface with the coagulation system, whose effector compartment (tissue factor, thrombin, platelets, neutrophils, complement) overlaps extensively with the pathways modulated by MSC-derived signals.

Available evidence indicates that MSCs can directly modulate coagulation. Most experimental studies report a predominantly procoagulant activity, particularly after intravenous administration and at higher doses [8,9,13,14,15]. This effect is largely mediated by tissue factor (TF/CD142) expression and by phosphatidylserine exposure on the cell surface and on extracellular vesicles, which activates the extrinsic pathway and thrombin generation [8,9,13,14,16]. Under whole-blood conditions, MSCs and their derivatives may also trigger an instant blood-mediated inflammatory reaction (IBMIR). IBMIR denotes an immediate reaction occurring within minutes of cell–blood contact, in which coagulation, complement, and leukocyte responses activate simultaneously on the surface of the infused cell population; the phenomenon was originally described in pancreatic islet transplantation, where it accounted for rapid loss of transplanted islets despite their viability at the time of infusion [8,9].

Counter-regulatory mechanisms coexist with this procoagulant profile. In vitro and animal studies describe anti-platelet activity mediated by CD73-dependent adenosine production and the expression of factors capable of attenuating coagulation propagation [15,17]. The net effect reflects the interaction between these opposing inputs and is shaped by cell source, dose, and product characteristics, with the quantitative picture developed in the following sections.

MSCs should therefore not be interpreted as cells with either pro- or anticoagulant properties. They behave as biologically active components within a broader network where coagulation and innate immunity converge, acting as dynamic modulators of the immunothrombotic axis.

## 3. Tissue Factor Expression: Constitutive or Inducible?

TF expression in MSCs cannot be interpreted as a binary phenomenon of presence or absence. Evidence indicates the coexistence of a constitutive component, strongly influenced by tissue source, with a dynamic modulation driven by environmental stimuli and culture conditions.

Most of these observations derive from in vitro studies comparing MSC sources under defined expansion conditions. MSCs derived from adipose tissue, umbilical cord, or placenta frequently display relatively high levels of TF/CD142 even under standard expansion conditions. Bone marrow-derived MSCs generally exhibit a TF-low profile, sometimes with predominantly intracellular localization and limited surface exposure [10,16,18]. This pattern suggests a basal, partially cryptic form that may become functionally active under specific conditions.

Alongside the constitutive component, TF expression appears clearly inducible. Exposure to pro-inflammatory cytokines such as TNF-α, IFN-γ, and IL-1β increases surface TF in both bone marrow- and adipose-derived MSCs. This upregulation occurs without loss of the stromal phenotype and may coincide with enhanced immunomodulatory activity [16,19]. Hypoxic conditions also appear to influence TF expression. Culture at low oxygen tension (approximately 2% O_2_) has been associated with increased TF levels in MSCs derived from umbilical cord and dental pulp [16]. Technical variables, including cell density, passage number, and culture medium composition, can progressively modify TF expression and the overall procoagulant activity of MSC products [16].

This procoagulant phenotype should therefore not be viewed as a fixed cellular property but as a modulable determinant shaped by tissue origin, manufacturing conditions, and cryopreservation history. The thrombotic potential of MSC administration may not reflect an intrinsic property of the cells alone but emerge from the interaction between baseline cellular biology and the inflammatory or technical context in which cells are produced and administered. Clinical validation of these source- and culture-dependent differences remains limited.

The molecular basis of source-related differences in TF expression has not been fully resolved in MSC biology, but converging evidence from other cell systems provides a plausible framework. The human F3 promoter contains functional binding sites for AP-1 (c-Fos/c-Jun), NF-κB/Rel, Sp1, and Egr-1, and its inducible activity depends on cooperative engagement of these elements [20]. Lipopolysaccharide and proinflammatory cytokines activate TF transcription in monocytic cells through AP-1 and NF-κB, whereas hypoxia and mechanical stress recruit Egr-1 as the dominant driver. Whether the higher constitutive TF levels observed in adipose- and umbilical cord–derived MSCs reflect baseline activity of these transcription factors, permissive chromatin states at the F3 locus, or differences in promoter methylation remains to be formally addressed. Comparative epigenomic analyses of TF-low and TF-high MSC sources are not yet available. This gap contributes to the current difficulty in predicting procoagulant behavior from phenotypic marker panels.

Cryopreservation represents a clinically important but incompletely characterized determinant of this profile. Most commercially produced MSC batches are administered as cryopreserved products thawed immediately before infusion. In adipose-derived MSCs, thawing upregulates surface expression of this upstream trigger and increases procoagulant activity compared with continuously cultured cells, with the increase measurable within hours [21]. Cryopreservation also disrupts the actin cytoskeleton and compromises fibronectin-mediated endothelial adhesion, which correlates with reduced in vivo pulmonary retention after intravenous delivery [22]. The thawed product that reaches the patient is not biologically equivalent to the fresh cell used in most preclinical hemocompatibility studies. A post-thaw recovery step, or an IFN-γ pre-licensing step, has been proposed as partial mitigation, but neither has been formally integrated into release criteria.

## 4. Tissue Factor, Thrombin Generation, and Functional Hemocompatibility Assessment

Inflammation-induced upregulation of tissue factor does not always translate into a proportional increase in thrombin generation. In controlled in vitro systems such as macrophages, endothelial cells, or activated T lymphocytes, cytokine-induced TF expression is typically accompanied by measurable increases in procoagulant activity and thrombin generation, often with a clear dependence on factor VIIa [16,23,24,25]. Similar observations apply to mesenchymal stromal cells. Functional inhibition or silencing of tissue factor reduces markers of thrombin activation in ex vivo and in vivo experimental models, supporting the biological relevance of the TF–FVIIa axis in MSC-associated coagulation responses [26,27].

In more complex biological or clinical settings, the relationship between the proportion of TF-positive cells and the magnitude of the coagulation response appears less linear. In clinical cohorts treated with umbilical cord–derived MSCs, relatively high frequencies of TF-positive cells do not necessarily correspond to measurable changes in D-dimer levels, fibrinogen concentrations, or standard coagulation times [16]. Soluble TF released by endothelial cells stimulated with TNF-α or IL-6 becomes procoagulant only after adequate relipidation with phospholipids. In the absence of an appropriate membrane context, its functional activity remains minimal [28].

Phenotypic expression alone is therefore not a sufficient biomarker for predicting thrombin generation. Several factors govern whether the upstream signal becomes functionally active within the coagulation cascade. These include TF “decryption”, exposure of phosphatidylserine, availability of circulating FVIIa and FX, and the co-regulation of anticoagulant pathways such as TF pathway inhibitor (TFPI), thrombomodulin, and the endothelial protein C receptor (EPCR). Extracellular vesicles, complement activation, and platelet responses further modulate the final thrombin output [16,24,28,29,30].

This dissociation is the operational rationale for functional hemocompatibility testing. Marker-based characterization captures the upstream signal. It does not capture whether that signal is competent to generate thrombin under the specific membrane, protein, and flow conditions of the product at infusion. A functional assay panel is therefore required to complement phenotype, particularly when the intended route of administration is intravascular [10,11].

Among functional assays, calibrated automated thrombography, commonly referred to as the thrombin generation assay (CAT/TGA), is widely used to quantify the thrombin-generating capacity of MSCs or their extracellular vesicles. Parameters such as lag time, peak thrombin, and endogenous thrombin potential (ETP) provide a direct estimate of the thrombogenic potential associated with the cellular product [9,10]. Whole-blood viscoelastic tests such as thromboelastography (TEG) or rotational thromboelastometry (ROTEM) capture the dynamics of clot initiation and formation in a more physiologically integrated environment. These assays reveal differences related to cellular source, culture conditions, or product preparation [10,16]. Whole-blood circulation models such as the Chandler loop system allow simultaneous evaluation of platelet consumption, microthrombus formation, and activation of inflammatory pathways including IBMIR [10,11]. TF expression is commonly assessed by flow cytometry, measuring both the proportion of TF-positive cells and fluorescence intensity, and may also be evaluated on extracellular vesicles. Western blotting or ELISA are sometimes used to quantify total or soluble TF [10,16,31].

A further unresolved issue concerns standardisation. No universally accepted thresholds of CD142 expression or standardised parameters of thrombin generation or viscoelastic assays exist for use as release criteria for MSC products intended for intravascular administration. Current characterisation protocols report CD142 with heterogeneous antibodies, cytometric gating strategies, and reference cell lines. No consensus threshold distinguishes a hemocompatible from a thrombogenic MSC batch. A similar heterogeneity applies to thrombin generation assays, where pre-analytical variables such as platelet-poor versus platelet-rich plasma, tissue factor reagent concentration, and cell-to-plasma ratios influence the output. Harmonisation of these parameters, ideally through a shared reference framework endorsed by regulatory bodies and manufacturers, is a prerequisite for moving hemocompatibility testing from a research tool toward a formal release criterion [10,11].

Within the working biphasic framework proposed in this review, tissue factor expression and phosphatidylserine availability represent the biochemical core of the early activation phase. Tissue factor provides the upstream trigger for extrinsic-pathway activation, whereas phosphatidylserine supplies the anionic membrane surface required for assembly of coagulation complexes and amplification of thrombin generation. Their combined activity helps determine whether MSC–blood contact remains a limited biological signal or progresses toward a clinically relevant procoagulant response.

## 5. Platelets as a Central Hub in MSC-Driven Immunothrombosis

Beyond the upstream coagulation trigger, MSC interaction with circulating platelets represents an additional determinant of the coagulation response. Evidence remains heterogeneous and derives from a combination of in vitro studies and experimental models. Platelets are a central node at the interface between innate immunity and coagulation, and their interaction with MSCs suggests a bidirectional regulatory dynamic that extends beyond the TF–thrombin axis.

One of the best characterized inhibitory mechanisms involves the CD73–adenosine pathway. Netsch et al. demonstrated that MSCs derived from both bone marrow and lipoaspirate inhibit platelet activation and aggregation in vitro through adenosine generated by the ectonucleotidase activity of CD73 [17]. Functional analyses of platelet reactivity confirmed that MSCs can directly modulate primary hemostasis through this pathway. The interaction between MSCs and the megakaryocytic compartment may not be unidirectional. In experimental models, mitochondrial transfer from megakaryocytes to MSCs through connexin-43-mediated gap junctions has been associated with reduced platelet activation and with a metabolic profile compatible with a functionally quiescent state [32]. These findings indicate that MSC-mediated platelet regulation may involve metabolic and stromal mechanisms acting upstream of intravascular platelet activation.

Expression of tissue factor, the primary activator of the extrinsic coagulation pathway, remains the dominant procoagulant input under conditions of direct blood exposure [33]. Activation of the coagulation cascade may also translate into secondary platelet activation through thrombin generation. The coexistence of an inhibitory axis (CD73–adenosine) and a procoagulant axis (extrinsic pathway activation and thrombin generation) therefore suggests a bidirectional regulatory framework whose outcome depends on the inflammatory microenvironment and on cellular production conditions.

Bidirectionality is further supported by evidence that platelets themselves can modulate MSC function. Vogel et al. reported that activated platelets inhibit the recruitment of MSCs toward injured cardiomyocytes through a mechanism involving HMGB1/TLR-4 signalling and downregulation of the MET receptor for hepatocyte growth factor (HGF) [34]. Platelets should not be viewed merely as downstream effectors of coagulation; they actively participate in regulating MSC migration and regenerative activity.

The MSC–platelet interaction represents a dynamic circuit in which primary hemostasis becomes an integration point linking tissue repair and immunothrombosis.

## 6. MSC-Derived Extracellular Vesicles: Subcellular Platforms of Coagulation Activation

Extracellular vesicles derived from mesenchymal stromal cells (MSC-EVs) represent a biologically active subcellular component that retains and reorganises part of the procoagulant potential of the parent cells. Current evidence on MSC-EVs is derived mainly from in vitro assays and preclinical models. Unlike intact MSCs, EVs do not provide a continuous cellular surface; they expose lipid microdomains enriched in phosphatidylserine and, in many cases, TF. These structures may function as high-density platforms for the assembly of coagulation complexes. In models using human blood, both MSCs and MSC-EVs accelerate thrombin generation in a dose-dependent manner, with measurable effects in thrombin generation assays and in dynamic whole-blood systems [13].

The procoagulant activity of MSC-EVs appears closely related to the functional presence of tissue factor on their surface. Inhibition of this pathway significantly reduces the thrombin-generating capacity of these vesicles, supporting a direct contribution of the upstream trigger to propagation of the coagulation cascade [35,36]. The biological effect of EVs may depend on vesicle size and molecular cargo. In a murine model, large EVs derived from umbilical cord MSCs induced pulmonary thromboembolism in a dose- and TF-dependent manner, demonstrating that EV-associated tissue factor remains functionally active in vivo [36]. EVs isolated from umbilical cord MSCs show procoagulant activity that varies with production conditions, suggesting that vesicle composition influences their thrombin-generating potential [31].

Compared with intact MSCs, EVs do not reproduce the same dynamics of pulmonary entrapment or phagocytosis-mediated clearance. They retain the intrinsic capacity to support thrombin generation through the combined presence of this upstream trigger and exposed anionic phospholipid surfaces. MSC-EVs should therefore not be considered neutral with respect to the immunity–coagulation interface. They represent a distinct mode of presenting procoagulant signals that is fragmented, spatially concentrated, and lacking the broader regulatory context provided by the intact cell. EVs may be viewed as a subcellular expression of the hemostatic potential of MSCs, capable of sustaining thrombin generation while not fully reproducing the complex immunological interactions associated with whole cells.

## 7. MSCs, Complement, and NETs: Integration Within the Immunothrombotic Axis

Complement engagement accompanies coagulation activation from the earliest phase of MSC–blood contact. When MSCs enter the intravascular compartment, surface-exposed carbohydrate and protein motifs bind C3 opsonins and trigger both classical and alternative pathways. Complement deposition proceeds in parallel with platelet adhesion, thrombin generation, and neutrophil recruitment, defining a coordinated immunothrombotic circuit rather than a sequence of independent injuries. Contact between MSCs and human blood rapidly activates the complement system, leading to deposition of C3b/iC3b and C3dg on the cellular surface and to the release of the anaphylatoxins C3a and C5a [37,38]. MSCs express relatively low levels of complement regulators such as CD46 and CD55 while retaining CD59, and become opsonized without undergoing extensive complement-mediated lysis. Opsonization promotes interaction with monocytes and other innate effector cells through complement receptor CR3 (CD11b/CD18) [37,38]. Complement activation also contributes to some of the immunomodulatory effects of MSCs, including the induction of regulatory myeloid populations. Pharmacological inhibition of complement or depletion of CD14^+^ monocytes reduces these effects, suggesting that complement-dependent opsonisation is a necessary step in MSC-mediated immune modulation [37].

MSCs also influence neutrophil responses. In models of myocardial ischemia–reperfusion injury, extracellular vesicles derived from MSCs reduce neutrophil infiltration and limit the formation of neutrophil extracellular traps (NETs). NETs are web-like structures composed of decondensed chromatin, histones, and granule-derived antimicrobial proteins (among them neutrophil elastase and myeloperoxidase) extruded by activated neutrophils; they trap and kill pathogens extracellularly and, in the same gesture, provide a negatively charged scaffold on which platelet adhesion, fibrin deposition, and thrombin generation are amplified, making NETs a direct mechanistic bridge between neutrophil activation and intravascular thrombosis. MSC-EV–mediated reduction of NET formation attenuates microvascular obstruction in these models, an effect associated with downregulation of the NLRP3 inflammasome and modulation of specific microRNAs in neutrophils [39]. Additional experimental data indicate that MSCs can suppress NET formation in models of systemic inflammation, attenuating a central effector mechanism of immunothrombosis [8].

Complement activation induced by MSC infusion extends beyond a simple cytotoxic process and becomes part of a broader network linking plasma systems of innate immunity with coagulation pathways. Early interaction between infused MSCs and circulating blood components appears conceptually related to IBMIR, characterised by simultaneous activation of complement, coagulation, and cellular inflammatory pathways. Complement and coagulation share mechanisms of reciprocal activation. Thrombin can cleave C3 and C5, whereas C5a and the terminal complement complex promote TF expression on monocytes and endothelial cells [7,8].

Within cellular therapies, intravascular infusion of MSCs exposes a biologically active surface that may simultaneously activate complement, platelets, and the coagulation cascade [11,38]. Expression of tissue factor on MSCs represents a major determinant of thrombin activation, whereas complement-derived mediators amplify the procoagulant signal through anaphylatoxin release and myeloid cell activation.

These interactions also shape the fate of MSCs after infusion. Clearance should therefore not be interpreted solely as an immunological process but as an event occurring at the interface between hemostasis and innate immunity. Phagocytosis of apoptotic MSCs and the development of regulatory monocyte phenotypes occur within a plasma environment already influenced by thromboinflammatory signals. Under physiological conditions or with relatively low cellular doses, this response may remain controlled. High cellular load, pre-existing inflammation, or increased TF expression may shift the balance toward a more pronounced procoagulant response.

These observations suggest a dynamic working framework in which early procoagulant activation and complement engagement may be followed by immune remodelling that attenuates inflammation and indirectly limits propagation of immunothrombosis [3,6,11]. MSCs should not be viewed solely as triggers of thrombosis or exclusively as anti-inflammatory agents. They act as modulators of central nodes within the immunity–coagulation axis, and the final outcome, pro- or antithrombotic, depends on the balance between early activation events (the upstream procoagulant trigger, IBMIR, and complement) and subsequent reorganisation of the myeloid and neutrophil compartments.

This balance represents the transition point between the two phases proposed in this review. In the early phase, MSC–blood contact may activate coagulation, complement, platelets, and neutrophil-dependent amplification pathways. In the subsequent phase, the same interaction may contribute to immune regulation if complement deposition, cellular injury, and phagocytic clearance remain controlled and lead to myeloid reprogramming rather than sustained thromboinflammatory propagation.

**Figure 1:**
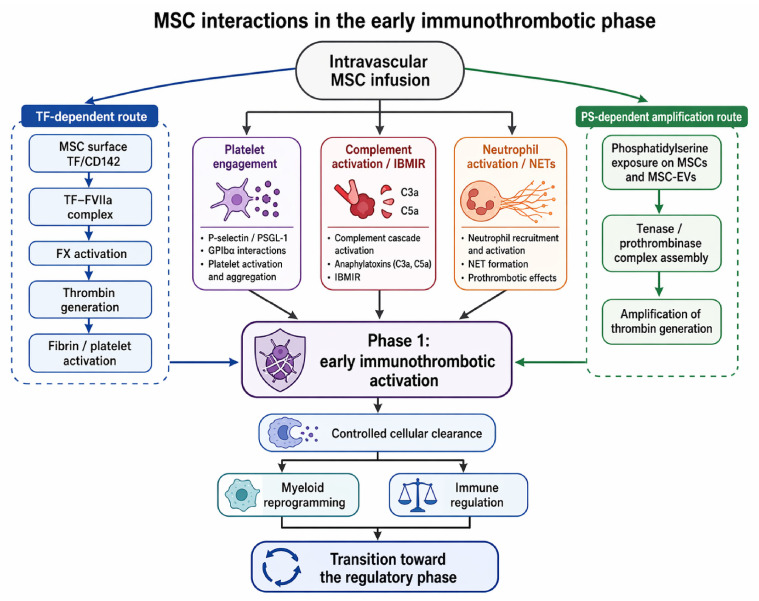
MSC interactions in the early immunothrombotic phase. Following intravascular administration, MSCs may promote coagulation through two partially distinct but convergent routes. Tissue factor/CD142 provides the upstream trigger for extrinsic-pathway activation through the TF–FVIIa axis, whereas phosphatidylserine exposure on MSCs and MSC-derived extracellular vesicles supports tenase and prothrombinase complex assembly and amplifies thrombin generation. Platelet engagement, complement activation, IBMIR, and neutrophil/NET responses further reinforce this early Phase 1 signal. Controlled cellular clearance and myeloid reprogramming then provide the transition toward the regulatory phase represented in the proposed buphasic framework.

These early interactions are summarised in Figure 1, with explicit separation between tissue factor-dependent coagulation initiation and phosphatidylserine-dependent amplification of thrombin generation.

## 8. Complement, Clearance, and “Cell Sacrifice” Hypothesis: The Paradox of MSC Fate After Intravenous Infusion

The early interactions with plasma systems also shape the subsequent fate of infused MSCs within the circulation. Activation of complement and innate immune pathways has substantive consequences for the fate of the infused cells. Intravenous infusion of MSCs does not result in long-term cellular engraftment within target tissues. It leads to an immediate and intense interaction with the circulating blood compartment. Contact with serum rapidly activates complement, resulting in deposition of C3b and formation of the membrane attack complex (MAC), which can induce dose-dependent cellular injury [40]. The phenomenon was initially interpreted as a limitation of MSC therapy. Its magnitude appears strongly influenced by clinical context. At commonly used doses (approximately 1–3 × 10^6^ cells/kg), systemic complement activation generally remains limited and is not associated with clinically relevant markers of coagulopathy [41].

Most infused MSCs become rapidly trapped within the pulmonary microcirculation, where their half-life is estimated to be on the order of 24 h [42]. During this period many cells undergo apoptosis or are phagocytosed by circulating monocytes and resident macrophages. Studies in murine models and in patients with graft-versus-host disease indicate that phagocytosis of apoptotic MSCs does not represent therapeutic failure. It appears to constitute a critical step in their mechanism of action. Myeloid cells that engulf apoptotic MSCs acquire an immunoregulatory phenotype characterised by increased IL-10 production, upregulation of PD-L1, and induction of regulatory T cells [43,44]. Generation of indoleamine-2,3-dioxygenase (IDO) by phagocytic cells seems to play a central role in the systemic immunosuppressive response [44].

These observations suggest a shift in perspective. Therapeutic efficacy of MSCs may depend less on cellular persistence or engraftment than on dynamic interaction with complement and myeloid cells. Even transient pulmonary residence may serve a functional purpose. Cells trapped in the pulmonary circulation have been shown to increase the expression of anti-inflammatory mediators such as TSG-6 before undergoing clearance [42]. What initially appeared to be rapid elimination may represent an integral component of the therapeutic cascade.

Complement activation and phagocytosis should not be viewed solely as obstacles to cellular survival. They help define the temporal window within which MSCs exert their biological activity. Excessive complement activation may reduce cell viability and secretory function, whereas insufficient cytotoxic activity in the host could limit efferocytic pathways and attenuate the downstream immunomodulatory response. The intravascular fate of MSCs thus appears to lie at the intersection of cellular injury, immune signaling, and regulatory activation. Therapeutic effects may ultimately depend on a finely balanced equilibrium between transient survival and functional cellular sacrifice.

Early complement and coagulation activation following MSC–blood contact should not be interpreted exclusively as a safety concern. It may also represent a determinant of the biological fate and mechanism of action of infused cells. Opsonization, interaction with myeloid cells, and rapid clearance do not necessarily signify therapeutic failure; they may constitute functional steps that initiate downstream immunoregulatory cascades. Hemocompatibility and immunomodulation therefore appear as interconnected outcomes of the same initial interaction between MSC products and the intravascular environment [10].

## 9. Contextual Duality, Biphasic Framework, and Testable Predictions

Mesenchymal stromal cells do not behave as biologically neutral elements once exposed to blood. Experimental evidence indicates that they can display both anti-thrombotic and procoagulant activities. The direction of the net effect appears to depend on biological context, tissue source of the cells, and conditions used during ex vivo expansion [17,18,45,46,47].

On the inhibitory side, the CD73–adenosine axis described in Section 5 and the suppression of neutrophil-driven immunothrombosis exemplify a direct de-thromboinflammatory effect. In models of lipopolysaccharide-induced acute lung injury, MSC administration reduces NET formation and improves survival, consistent with this function [46].

On the activating side, experimental studies consistently show that many MSC preparations possess intrinsic procoagulant activity, largely related to expression of the upstream trigger and phosphatidylserine exposure [13,18,45,47]. Adipose-derived MSCs often exhibit a more procoagulant profile than bone marrow-derived MSCs, and the magnitude of the effect may vary with culture duration and manufacturing procedures [18].

Evidence from animal models further supports the functional relevance of the procoagulant arm. Under certain experimental conditions, coagulation activation driven by this pathway after MSC infusion has been associated with severe thromboembolic events [45]. Route of administration also matters: intracoronary delivery in porcine myocardial infarction models produces microvascular obstruction and microthrombosis, effects that could be partially attenuated by antithrombin therapy such as heparin [47].

In vitro hemocompatibility studies confirm this dual behaviour. Both MSCs and their EVs accelerate clot formation in functional assays such as ROTEM or thrombodynamics systems, and anticoagulation with heparin reduces but does not always abolish the procoagulant effect [13].

Recent reviews converge on a key interpretative point: the apparent duality of MSC effects should not be read as a contradiction, but as a biological phenomenon that requires quantification and control. In many settings, the procoagulant component is associated with technical determinants such as tissue source, culture conditions, dose, and route of administration, rather than with an unavoidable property of MSC therapy [8,9].

### 9.1. Regulatory T Cells and Thrombus Resolution: A Speculative Extension

A speculative extension of this framework concerns regulatory T cells (Tregs). Two independent bodies of evidence motivate the hypothesis but do not intersect experimentally. Tregs participate in the resolution phase of venous thrombosis in murine models: a specialised thrombus-infiltrating subset producing the matricellular protein SPARC accumulates within the clot and the surrounding vein wall and supports monocyte recruitment and matrix metalloproteinase activity, facilitating fibrin and matrix degradation [48]. Depletion of CD4 and CD8 lymphocytes impairs venous thrombus resolution, with reduced macrophage accumulation and lower expression of fibrinolytic mediators [49,50]. In parallel, MSCs promote Treg expansion in multiple experimental settings unrelated to thrombosis, including graft-versus-host disease and chronic inflammatory conditions [51]. No study has directly demonstrated that MSC-induced Treg expansion mediates thrombus resolution in vivo. The chain MSC → Treg expansion → thrombus resolution is therefore a conjectural extension constructed by concatenating two independent lines of work rather than a substantiated pathway. It is retained as a testable hypothesis because the logical connection is plausible and worth testing, not because it has been verified. The experimental programme required to address it is outlined in Section 9.3.

### 9.2. Biphasic Framework: An Interpretative Hypothesis

**Figure 2:**
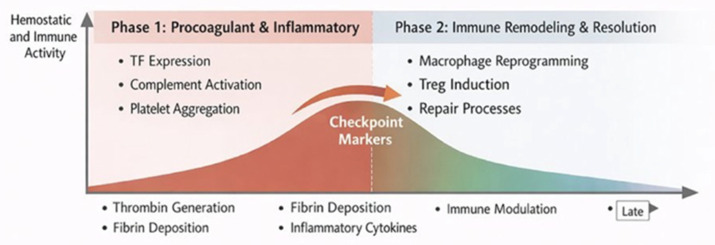
Proposed biphasic framework model of MSC infusion response. Phase 1 is characterized by extrinsic-pathway activation, complement activation, and platelet aggregation, generating an early procoagulant and inflammatory signal. Transition through the checkpoint zone, marked by peak D-dimer, rising Treg frequency, and macrophage polarization shift, initiates Phase 2, in which immune remodeling, Treg induction, and repair processes progressively attenuate the immunothrombotic response. Conditions that sustain high cell dose or host inflammation may delay or prevent Phase 2 entry.

Taken as a whole, these observations support an interpretative biphasic framework rather than a demonstrated sequence. Early intravascular exposure appears to be dominated by procoagulant and complement-dependent activation. Later phases may favor immune regulation through macrophage reprogramming and, speculatively, Treg expansion. The timing and strength of this transition are likely shaped by cell dose, host inflammatory tone, and the efficiency of apoptotic clearance as summarised in the proposed biphasic framework shown in Figure 2.

### 9.3. Testable Predictions and Proposed Experimental Tests

The working framework yields a limited set of falsifiable predictions that can be addressed with focused preclinical and early translational studies.

**H1.**  *Dose dependence of phase transition. A graded-dose design should identify whether higher MSC doses prolong the procoagulant phase, with sustained elevation of D-dimer, thrombin–antithrombin complexes, or prothrombin fragment 1 + 2 beyond the transient changes expected at standard doses.*

**H2.**  *Recipient inflammation delays phase 2. In endotoxemic or sepsis-primed models, the same MSC dose should produce stronger coagulation activation and weaker late immune-remodeling signatures than in non-inflamed controls; the Perlee human endotoxemia model provides a relevant reference platform [26].*

**H3.**  *Cryopreservation shifts the balance toward phase 1. Matched fresh versus thawed products from the same donor line should clarify whether cryopreservation amplifies the early procoagulant signal without a proportional increase in macrophage reprogramming or Treg-associated readouts.*

**H4.**  *Ex vivo bioreactor delivery decouples the two phases. Comparative intravenous versus bioreactor studies should test whether preventing direct cell–blood contact attenuates early thrombin generation while preserving downstream immunomodulatory signatures [52,53].*

**H5.**  *Treg induction and clinical resolution. In exploratory human studies, peripheral Treg frequency measured 7–14 days after infusion could be correlated with markers of clinical resolution; any signal would be supportive rather than confirmatory.*

Negative results in these experiments would narrow the framework to its stronger mechanistic core-early coagulation activation, complement engagement, cellular clearance, and macrophage reprogramming-and would determine whether the Treg extension should be retained or abandoned.

## 10. Clinical Relevance of the Procoagulant Potential of MSCs

The pronounced procoagulant potential of MSCs observed in vitro has not, based on current evidence, translated into a clear increase in clinically significant thrombotic events in controlled human studies. Two large meta-analyses evaluating intravascular administration of MSCs, including more than 3000 patients overall, did not detect a significant increase in thromboembolic events compared with control groups. The overall incidence remained low and was similarly distributed between treated and placebo arms [54,55]. Consistent findings have been reported in randomized trials conducted in acute respiratory distress syndrome (ARDS) and COVID-19, and in other inflammatory or neurological indications, where the thrombotic safety profile of MSC therapy appears generally favorable [56,57,58,59].

Signals of subclinical coagulation activation are nevertheless frequently reported. Transient increases in D-dimer levels, thrombin-antithrombin complexes, or mild reductions in platelet counts have been described after MSC infusion, even in the absence of overt thrombotic events [54,60]. In high-risk contexts, such as portal infusion in metabolic liver diseases or combined transplantation procedures, thrombotic episodes have occasionally been observed, although these events were generally manageable with targeted anticoagulation strategies [60]. The clinical thrombotic risk is not absent, but depends on cell dose, route of administration, tissue source, and the tissue factor profile of the MSC product.

The divergence between preclinical procoagulant signals and clinical outcomes likely reflects patient selection, moderate dosing, standard thromboprophylaxis, and manufacturing improvements. Coagulation monitoring remains advisable in patients with active inflammation or pre-existing thrombotic risk.

The apparent safety profile derived from aggregate meta-analyses requires qualification when specific subgroups are considered. In patients with active systemic inflammation, MSC infusion can amplify procoagulant signalling rather than attenuate it. In a randomised human endotoxemia model, adipose-derived MSCs at 4 × 10^6^ cells/kg enhanced coagulation activation and reduced the fibrinolytic response compared with placebo [26]. In COVID-19 patients, who frequently present with hypercoagulable states and endothelial activation, the risk profile of intravenous MSC delivery depends on cell product quality and on baseline thromboprophylaxis [61]. Dose is an independent determinant. Preclinical work has shown that intravenous MSCs induce a dose-dependent coagulopathy with disseminated thrombosis at the highest doses, an effect that can be mitigated by heparin co-administration [15,62]. Clinical case reports provide complementary evidence. A family cluster of pulmonary embolism and pulmonary infarcts was reported after repeated intravenous infusion of autologous adipose-derived MSCs, with spontaneous resolution on follow-up imaging [63]. Hepatic routes introduce a distinct set of concerns. Intraportal infusion of liver-derived MSCs in patients with Crigler-Najjar syndrome and urea cycle disorders was associated with one partial portal vein thrombus, reversible platelet decrease, and D-dimer rise, requiring combined heparin and bivalirudin prophylaxis [60]. These subgroup-specific data indicate that the favorable aggregate safety signal should not be extrapolated to patients with pre-existing procoagulant states, to high-dose protocols, to non-peripheral delivery routes, or to combined-transplant scenarios without dedicated monitoring.

Most trials included in the safety meta-analyses were not designed with hemostatic endpoints as primary or secondary outcomes. Coagulation monitoring was often limited to routine parameters, collected at sparse time points, and subclinical activation markers such as thrombin–antithrombin complexes, prothrombin fragment 1 + 2, D-dimer kinetics, platelet activation markers, or viscoelastic indices were inconsistently reported. In addition, many studies were underpowered to detect uncommon thrombotic events and included heterogeneous products, routes of administration, doses, and thromboprophylaxis protocols. The reassuring aggregate data on major thrombotic events should therefore be interpreted as evidence against a large, uniform clinical safety signal, not as proof that MSC-associated coagulation activation is clinically irrelevant. Future trials would benefit from pre-specified coagulation endpoints, standardised post-infusion sampling windows, and extended follow-up in high-risk populations.

## 11. Determinants and Mitigation Strategies for Coagulation Risk

The coagulation risk associated with MSC therapy appears multifactorial and potentially modifiable. Tissue source is one of the main determinants, as summarized in Table 1 and developed in Section 3: bone marrow-derived MSCs generally display a TF-low phenotype with lower procoagulant activity, whereas adipose-, umbilical cord-, or decidua-derived cells more frequently show higher TF expression and stronger thrombin activation in vitro and in animal models [16,64]. Extracellular vesicles derived from MSCs contribute further to this effect. Vesicles originating from perinatal or adipose sources can exhibit marked and dose-dependent procoagulant activity, often mediated by the combined presence of tissue factor and exposed phosphatidylserine [13,14,36]. Phosphatidylserine provides the negatively charged phospholipid surface required for assembly of coagulation complexes, facilitating thrombin generation in the presence of TF.

Pharmacological mitigation has been explored as a complementary strategy. Heparin remains the most extensively studied approach. In a porcine model of myocardial infarction, co-administration of heparin during intracoronary MSC infusion reduced microvascular thrombosis and improved cardiac function compared with infusion without anticoagulation [47]. Preclinical studies further indicate that anticoagulation can attenuate MSC-induced procoagulant activity and reduce microvascular thrombus formation in models of intravascular administration [14]. Bivalirudin has been proposed as an alternative, and in a small clinical experience the combination of heparin and bivalirudin limited thrombogenic responses largely to subclinical changes in most patients receiving intravascular MSC therapy [9]. Periprocedural anticoagulation may not completely eliminate coagulation activation but can maintain it within clinically manageable limits.

This point is particularly relevant in light of the hypothetical cell sacrifice mechanism discussed above. If complement opsonisation, controlled cellular injury, and efferocytic clearance contribute to downstream immunomodulation, mitigation should not be interpreted as complete suppression of MSC–blood interaction. Its objective is more limited: to prevent uncontrolled propagation of coagulation while preserving, when possible, the early biological signals that may support clearance-driven immune regulation. Whether anticoagulant or complement-modulating strategies can alter this balance remains insufficiently defined and requires direct experimental evaluation.

Product characterisation is a second axis of mitigation, treated in detail in Section 4. Integration of CD142 expression and functional hemocompatibility assessment into release criteria for MSC products intended for intravascular use has been proposed on safety grounds [65]. The rationale rests on the phenotype–function dissociation developed there. Functional assays such as thromboelastometry or thrombin generation testing, combined with marker-based characterisation, represent a concrete step toward safety-oriented standardisation [14]. Variability between studies may also reflect differences in manufacturing protocols, including culture conditions, passage number, and cryopreservation procedures, which influence the surface phenotype and hemocompatibility profile of MSC products.

Dose and infusion dynamics represent additional critical variables. Preclinical studies show a dose–response relationship between the number of infused cells and thrombin generation, with dose-dependent coagulopathy and thromboembolic phenomena observed at high cell doses [14,15]. Lower doses and slower infusion rates are generally associated with reduced coagulation activation. Route of administration plays a substantial role. Rapid intravascular delivery appears to carry the highest risk, whereas local or non-intravenous routes reduce the immediate interaction with the hemostatic compartment [9,65].

Despite the pronounced procoagulant potential observed in experimental systems, systematic analyses of clinical data indicate that the incidence of major thrombotic events after intravascular MSC infusion remains relatively low in published trials [36]. The apparent discrepancy suggests that thrombogenicity depends largely on dose, route of administration, and the inflammatory status of the recipient, and may therefore be manageable through a combination of product selection and pharmacological modulation.

If the critical interface underlying coagulation activation is the direct interaction between MSCs and circulating blood components, an alternative strategy is to modify that interface rather than the cellular product itself. Approaches based on ex vivo systems or bioreactor platforms may preserve immunomodulatory signaling while reducing immediate intravascular activation of coagulation and complement. In this view, the mode of administration is not only a technical variable but also a biological determinant of the immunothrombotic response.

An ex vivo bioreactor delivery system targets this interface directly. MSCs are immobilised on the extraluminal side of hollow-fibre cartridges through which patient blood is perfused, allowing paracrine exchange with the intravascular compartment while preventing direct cell–blood contact. In preclinical models, the approach reduced clot formation relative to free MSC infusion and preserved target activated clotting time levels without requiring additional heparin [52]. A clinical application has been tested in patients with severe acute kidney injury and systemic inflammation using an allogeneic MSC bioreactor product, with a pharmacodynamic profile consistent with immunomodulation and without the thrombotic signal associated with intravascular cell exposure [53]. Whether this delivery mode can preserve efficacy across indications that have historically relied on cell homing and local engraftment remains to be determined, but it usefully reframes hemocompatibility as a design parameter rather than as a constraint to be managed after cell release.

**Figure 3:**
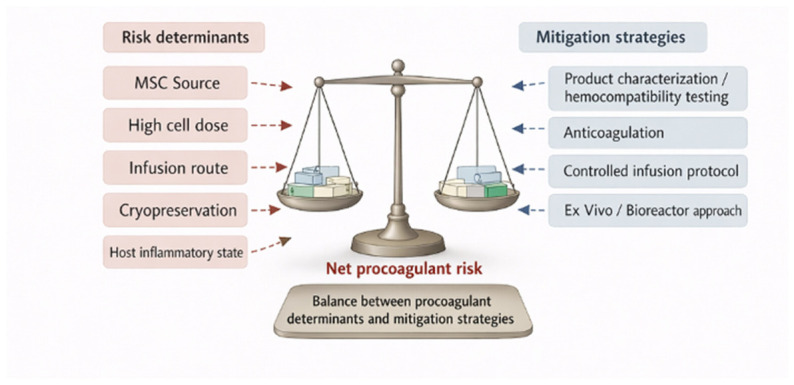
Determinants of procoagulant risk and mitigation strategies. Left panel: the principal risk determinants (MSC source, cell dose, infusion route, cryopreservation status, and host inflammatory state) tilt the balance toward net procoagulant risk. Right panel: the corresponding mitigation strategies (product characterization with hemocompatibility testing, anticoagulation, controlled infusion protocols, and ex vivo bioreactor delivery) counterbalance these risks. The clinical objective is not the elimination of all early MSC–blood responses, but their measurement and controlled modulation.

Thrombotic risk therefore emerges from the interplay of cell source, product characteristics, route of delivery, and recipient state. The main determinants of risk and the corresponding mitigation strategies are summarised in Figure 3, whereas Table 2 provides an illustrative source–route–context matrix to support risk stratification and prophylactic planning. 

## 12. Conclusions

The evidence discussed in this review indicates that MSCs should not be regarded as passive elements within the hemostatic environment. They behave as biologically active interfaces capable of modulating coagulation through several interconnected pathways. Tissue factor represents a major determinant of MSC-associated thrombogenicity, yet its expression alone does not fully account for the variability observed across experimental systems and clinical studies. The dissociation has a practical consequence: marker-based quantification alone, without functional assessment of thrombin generation and platelet engagement, is insufficient as a safety release criterion. Phenotypic characterisation should therefore be interpreted as one component of a broader hemocompatibility panel rather than as an independent predictor of thrombotic risk.

From a clinical perspective, the marked procoagulant potential documented in vitro has not consistently translated into a clear increase in major thrombotic events in controlled trials of intravascular MSC administration. Transient activation of coagulation pathways and occasional thrombotic complications in selected high-risk settings nevertheless indicate that the hemostatic profile of MSC therapy is context dependent. Cell dose, route of delivery, tissue origin, and the inflammatory status of the recipient are critical variables. Thrombogenicity is therefore better interpreted as an emergent feature of the interaction between infused MSCs and the host vascular environment than as an intrinsic property of the cellular product.

**Figure 4:**
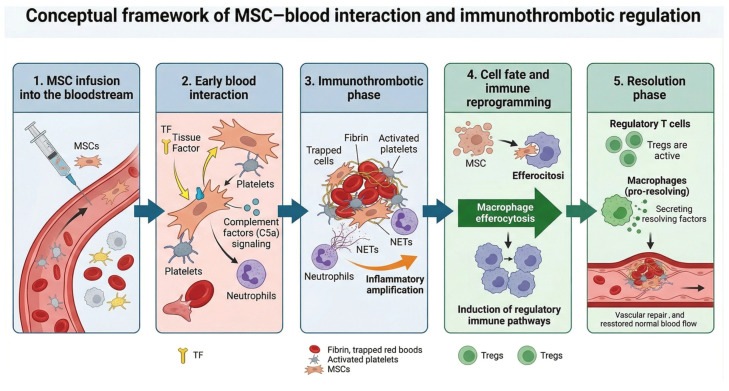
Conceptual framework of MSC–blood interaction and immunothrombotic regulation. Synthesis of the proposed biphasic response framework: (1) MSC infusion into the bloodstream; (2) early blood interaction through tissue factor, platelets, complement (C5a) signalling, and neutrophils; (3) immunothrombotic phase with fibrin deposition, platelet activation, NET formation, and inflammatory amplification; (4) cell fate and immune reprogramming driven by efferocytosis and induction of regulatory immune pathways; (5) resolution phase mediated by regulatory T cells and pro-resolving macrophages, restoring vascular repair and normal blood flow.

MSC–blood interaction is a coordinated immunothrombotic process in which early coagulation, complement, platelet, and neutrophil responses shape both immediate hemostasis and the subsequent fate of infused cells. Clearance through macrophage-mediated efferocytosis may initiate downstream regulatory programmes; Treg involvement in resolution (Section 9) remains conjectural and awaits the experimental programme proposed there. Translation will rest on rigorous product characterisation, functional hemocompatibility testing, and context-aware modulation of the vascular microenvironment. These relationships are summarised in the integrative conceptual framework shown in Figure 4.

## Figures and Tables

**Table 1:** Source-related variability in MSC hemocompatibility parameters. Values summarized from representative preclinical studies; figures should be interpreted as ordinal rather than quantitative across heterogeneous assay platforms. NC = not fully characterized; PE = pulmonary embolism; PS = phosphatidylserine.

MSC Source	TF/CD142	PS Exposure	Thrombin Generation	Complement Sensitivity	Clinical Thrombotic Risk	Refs.
Bone marrow	Low	Low–mod	Low	Moderate	Low; transient D-dimer rise	[8,12,13,55]
Adipose	High	Mod–high	High	Moderate	Low–mod; PE case reports	[8,11,12,60,61]
Umbilical cord	High	Moderate	High	High	Moderate; dose-dependent in preclinical	[12,21,22]
Placenta/decidua	High	High	High	High	Moderate; higher than BM	[12,55]
Liver-derived	Moderate	NC	Moderate	Moderate	Portal vein thrombosis; heparin + bivalirudin	[54]
Dental pulp	Mod–high	NC	Moderate	NC	Limited clinical data	[12]

**Table 2:** Pragmatic framework for clinical interpretation of MSC-associated thrombotic risk. This table summarizes patterns emerging from preclinical, translational, and limited clinical data. It should be interpreted as an author-derived framework for risk stratification and mitigation rather than as a formal guideline or evidence-based treatment recommendation. Local protocols, regulatory requirements, and clinical judgment remain decisive. AD, adipose-derived; AKI, acute kidney injury; ARDS, acute respiratory distress syndrome; BM, bone marrow-derived; F1 + 2, prothrombin fragment 1 + 2; IV, intravenous; LMWH, low-molecular-weight heparin; TAT, thrombin–antithrombin complex; TEG, thromboelastography; UC, umbilical cord-derived; UFH, unfractionated heparin; VTE, venous thromboembolism.

MSC Source	Route	Host Context	Estimated Risk Profile	Possible Risk-Mitigation Approach	Refs.
BM (TF-low)	IV peripheral	No active systemic inflammation; standard dose (1–3 × 10^6^/kg)	Low	Routine clinical monitoring; consideration of baseline coagulation markers according to local practice	[38,49]
AD or UC (TF-high)	IV peripheral	No active systemic inflammation; standard dose	Low–moderate	Careful product characterisation; consideration of periprocedural anticoagulation and post-infusion coagulation monitoring in selected cases	[8,12,13]
Any TF-high source	IV peripheral	Active systemic inflammation (e.g., sepsis, ARDS, severe COVID-19)	Moderate	Individualised thromboprophylaxis strategy; dose reduction and extended coagulation monitoring may be considered in high-risk settings	[15,58,59]
Any source	IV peripheral	High dose (>4 × 10^6^/kg) or repeated administration	Moderate–high	Dose minimisation, closer laboratory surveillance, and consideration of anticoagulation in relation to overall bleeding/thrombotic risk	[11,59,60]
BM (TF-low)	Intracoronary	Post-infarct myocardium	Moderate	Anticoagulation during delivery appears advisable on the basis of preclinical evidence	[47]
Liver-derived	Intraportal	Paediatric metabolic liver disease	High	Intensive periprocedural anticoagulation and imaging/laboratory monitoring should be considered; combined heparin–bivalirudin has been reported in clinical practice	[54]
Cryopreserved–thawed products (any source)	IV peripheral	Standard dose, any host state	Potentially increased versus fresh products	Post-thaw recovery and closer hemocompatibility assessment may be considered; whether fresh products are preferable remains unresolved	[61,62]
Any source	Ex vivo bioreactor	Severe AKI or systemic inflammation	Potentially lower than direct IV exposure	Extracorporeal delivery may reduce direct cell–blood contact and could represent a lower-risk alternative in selected contexts	[63,64]

## Data Availability

No new data were created or analyzed in this study.
